# Predictors of persistent disease activity following anti-VEGF loading dose for nAMD patients in Singapore: the DIALS study

**DOI:** 10.1186/s12886-020-01582-y

**Published:** 2020-08-06

**Authors:** Colin S. Tan, Louis W. Lim, Wei Kiong Ngo, Pandiyan Pannirselvam, Clarence See, Wai Kitt Chee, Nakul Saxena

**Affiliations:** 1 Fundus Image Reading Centre, National Healthcare Group Eye Institute, Singapore, Singapore; 2 National Healthcare Group Eye Institute, Tan Tock Seng Hospital, 11 Jalan Tan Tock Seng, Singapore, 308433 Singapore; 3 Lee Kong Chian School of Medicine, Nanyang Technological University, Singapore, Singapore; 4 Duke-NUS Medical School, National University of Singapore, Singapore, Singapore; 5 Novartis (Singapore) Private Limited, Singapore, Singapore

**Keywords:** Anti-VEGF, Neovascular age related macular degeneration, Polypoidal choroidal vasculopathy, Persistent disease activity

## Abstract

**Background:**

To determine the frequency of persistent disease activity following 3 loading doses of anti- vascular endothelial growth factor (VEGF) agents, and the anatomic and demographic predictors of early persistent disease activity among patients with neovascular age-related macular degeneration (nAMD).

**Methods:**

In a retrospective real-world cohort study, 281 consecutive patients with nAMD were reviewed at baseline and after 3 anti-VEGF injections for pre-defined indicators of disease activity. Optical coherence tomography (OCT) features such as subretinal fluid, intraretinal cysts and intraretinal fluid were assessed by reading-center certified graders. Multiple logistic regression was performed on demographic and anatomic factors.

**Results:**

At month 3, 66.1% of patients had persistent disease activity. The best-corrected visual acuity (BCVA) improvement was 0.16 LogMAR for those with no disease activity compared to 0 for patients with persistent activity (*p* < 0.001). The significant risk factors for persistent activity at 3 months were male gender (odds ratio [OR] 0.54, 95% confidence interval [CI] 0.32–0.93, *p* = 0.025), intraretinal cysts at baseline (OR 2.95, 95% CI 1.67–5.20, *p* < 0.001) and subretinal fluid at baseline (OR 3.17, 95% CI 1.62–6.18, *p* = 0.002). At 3 months, 58% of patients had features of activity on OCT. Patients with intraretinal cysts and intraretinal fluid at baseline had worse BCVA at month 3 compared to patients without these OCT features (0.69 vs. 0.43, *p* < 0.001, and 0.62 vs. 0.43, *p <* 0.001, respectively).

**Conclusions:**

In a real-world study, 66.1% of nAMD patients have persistent disease activity after the initial loading dose, with poorer BCVA compared to those without. Baseline OCT features (intraretinal cysts and subretinal fluid) are useful predictors of persistent disease activity at month 3.

## Background

Age-related macular degeneration (AMD) is the leading cause of blindness among individuals older than 50 years in developed nations [1, 2], and may result in severe visual loss.

Advances in imaging technology, especially optical coherence tomography (OCT), have revolutionized AMD diagnosis and treatment algorithms [3]. Features such as intra-retinal fluid and sub-retinal fluid detected on OCT are assessed to determine disease activity and treatment response.

Studies have reported that the initial treatment response to anti- vascular endothelial growth factor (VEGF) agents may be predictive of the long-term clinical outcome [4, 5]. However, many randomized controlled clinical trials, as well as non-randomized, single-arm studies, typically report the main outcomes at 12 months. Few studies have focused on the early outcomes after the initial 3 loading doses of anti-VEGF. Another key knowledge gap is the factors which predict the initial, early response to treatment.

Determining the proportion of nAMD patients with persistent disease activity, and the predictors of persistent disease activity following the initial loading dose of anti-VEGF, will help clinicians identify potential high-risk patients for whom a more rigorous treatment and follow-up regimen can be established.

The objective of this study is to determine the proportion of patients with persistent disease activity, following 3 loading doses of the same anti-VEGF, and the demographic and anatomic predictors of persistent disease activity.

## Methods

In a single-centre retrospective cohort study, consecutive treatment-naive patients diagnosed with either nAMD or Polypoidal Choroidal Vasculopathy (PCV) at the National Healthcare Group Eye Institute, Tan Tock Seng Hospital between 1 January 2014 to 31 October 2017 were reviewed. Treatment outcomes were assessed after 3 monthly loading doses (defined as 3 injections within 90 days of the first injection) of anti-VEGF drugs (Ranibizumab, Bevacizumab or Aflibercept). Patients were reviewed within 4 weeks of the third anti-VEGF injection.

All patients underwent a spectral domain OCT scan (Spectralis, Heidelberg Engineering, Heidelberg, Germany) prior to and after receiving the 3 loading doses of anti-VEGF. On presentation, fluorescein angiography (FA) and indocyanine green angiography (ICGA) were performed on all patients to establish the diagnosis and to distinguish between nAMD and PCV [6–10].

Patients were excluded from the study if they received any treatment for nAMD or PCV prior to recruitment or if they underwent verteporfin photodynamic therapy. Patients were also excluded if cataract surgery was performed during the study period. Other disease conditions that may affect best-corrected visual acuity (BCVA) or central retinal thickness measurements, such as diabetic retinopathy, diabetic macular edema, retinal vein occlusion, epiretinal membrane and glaucoma, were also excluded.

The primary outcome measure was the proportion of patients with disease activity following 3 loading doses of anti-VEGF injections. Potential predictors of disease activity assessed were patient demographics, comorbidities, baseline BCVA, baseline OCT features and retinal thickness.

The study was approved by the institutional review board of the National Healthcare Group and conformed to the tenets of the Declaration of Helsinki.

Grading of retinal images were performed by trained graders from the Fundus Image Reading Center, National Healthcare Group Eye Institute, using standardized grading protocols. The diagnosis of PCV and its differentiation from nAMD was performed using the diagnostic criteria described in the EVEREST and EVEREST II studies [6–12]. Central subfield retinal thickness (CSFT) was measured using the proprietary Heidelberg viewer software [13–18]. Trained graders screened each OCT B-scan for segmentation errors and manually adjusted these when errors were present. The presence of intraretinal fluid, cysts and subretinal fluid was manually graded. Intraretinal cysts was defined as areas of round or oval hyporeflectivity with clear borders located within the retinal tissue. Intraretinal fluid was defined as widening of the retinal bands compared to normal, healthy retina. In our clinical practice, FA and ICGA are not routinely performed after the initial 3 treatments, unless there is a specific clinical indication, such as worsening of clinical findings.

Using both OCT and visual acuity criteria, we defined persistent disease activity as

1. Decrease in BCVA of ≥5 letters compared with baseline; or
2. Decrease in BCVA of ≥3 letters and CSFT increase ≥75 μm compared with baseline; or.
3. Any presence of intraretinal fluid / subretinal fluid / cysts at week 12.

Overall, 296 patients were screened, with 281 included for analysis. Of the 15 patients who were excluded, 13 patients had ungradable images, 1 had a diagnosis of cystoid macular oedema and 1 patient had no follow up scans at month 3.

Statistical analysis was performed using SPSS version 16 (SPSS Inc., Chicago, IL, USA). X^2^ tests were used to compare the proportions of various groups, and unpaired *t*-tests were used to compare means, with a *P* < 0.05 considered statistically significant. To account for various factors that may impact outcomes, multiple logistic regression was also performed.

## Results

The mean age of the 281 patients was 77.7 years (range, 54 to 98, SD ± 9.0), with 168 males (59.8%) and 113 females (40.2%) (Table 1). The majority of patients had nAMD (221 patients, 78.6%) while the remaining 60 (21.4%) were diagnosed with PCV. The proportion of anti-VEGF used was 60.1% bevacizumab, 27.8% ranibizumab and 12.1% aflibercept.

**Table 1:** Baseline characteristics of patients

	All patients
Age (years)	77.7 ± 9.0
Gender (%)	
Males	168 (59.8%)
Females	113 (40.2%)
Race (%)	
Chinese	264 (94.0%)
Malay	7 (2.5%)
Indian	5 (1.8%)
Others	5 (1.8%)
Diagnosis (%)	
Neovascular AMD	221 (78.6%)
PCV	60 (21.4%)
VA (ETDRS letters)	54.5 ± 40.5
Central retinal thickness (μm)	442.4 ± 192.9
Features of activity on OCT	
Intraretinal fluid	188 (66.9%)
Intraretinal cysts	135 (48.0%)
Subretinal fluid	229 (81.5%)
Pigment epithelial detachment	274 (97.5%)

*AMD* age related macular degeneration, *OCT* optical coherence tomography, *PCV* polypoidal choroidal vasculopathy, *VA* visual acuity, *ETDRS* Early Treatment Diabetic Retinopathy Study

On initial presentation, all patients had features of disease activity. At 3 months, after the initial 3 doses of anti-VEGF drugs, 179 patients (66.1%) were found to have persistent activity, based on the pre-defined criteria of OCT findings and / or changes in BCVA.

There were no significant differences in baseline demographics, BCVA or central retinal thickness between the patients with persistent disease activity at 3 months compared to those without disease activity at 3 months (Table 2).

**Table 2:** Comparison of baseline characteristics based on disease activity at month 3

	Persistent activity	No activity	*P* value
Age (years)	78.0 ± 8.3	77.1 ± 10.4	*P* = 0.459
Gender			*P* = 0.067
Male	114 (63.7%)	48 (52.2%)	
Female	65 (36.3%)	44 (47.8%)	
Race			*P* = 0.598
Chinese	169 (94.4%)	85 (92.4%)	
Malay	4 (2.2%)	3 (3.3%)	
Indian	4 (2.2%)	1 (1.1%)	
Others	2 (1.1%)	3 (3.3%)	
Diagnosis			*P* = 0.838
nAMD	142 (79.3%)	72 (78.3%)	
PCV	37 (20.7%)	20 (21.7%)	
VA (ETDRS letters)	53.5 ± 39.0	56.5 ± 41.0	*P* = 0.384
Central retinal thickness (μm)	458.5 ± 192.5	430.56 ± 189.5	*P* = 0.279
Intraretinal fluid			*P* = 0.626
Yes	122 (68.2%)	60 (65.2%)	
No	57 (31.8%)	32 (34.8%)	
Intraretinal cysts			*P* = 0.002
Yes	97 (54.2%)	32 (34.8%)	
No	82 (45.8%)	60 (65.2%)	
Subretinal fluid			*P* = 0.007
Yes	153 (85.5%)	66 (71.7%)	
No	26 (14.5%)	26 (28.3%)	
Pigment epithelial detachment			*P* = 0.412
Yes	174 (97.2%)	90 (97.8%)	
No	5 (2.8%)	2 (2.2%)	

The improvement in visual acuity and CSFT was greater among the group with no persistent activity compared to those with persistent disease activity. The mean visual acuity was 54.5 letters (Snellen equivalent of 6/24) at baseline and improved to 57.5 letters (Snellen equivalent of 6/18) at 3 months (*p* = 0.02). Among patients with no disease activity at month 3, the mean visual acuity (VA) improved by 8 letters, compared to 0.01 letters in patients with persistent disease activity (*p* < 0.001).

Overall, CSFT was 442.4 μm (range, 188 to 1534, SD ± 193.0), and decreased to 349.1 μm (range, 150 to 1534, SD ± 143.9) at month 3 (*p* < 0.001). Among patients with no disease activity at month 3, the mean CSFT improved by 93.0 μm, compared to 88.8 μm in eyes with persistent disease activity (*p* = 0.816).

### Risk factors for persistent disease activity

Univariate logistic regression showed that male gender, intraretinal cysts and subretinal fluid were predictive of disease activity at week 12 (Table 3). Performing stepwise multiple logistic regression analysis, the same factors were found to be predictive of persistent disease activity at 3 months: male gender (odds ratio [OR] 0.54, 95% confidence interval [CI] 0.32–0.93, *p* = 0.025), the presence of intraretinal cysts at baseline (OR 2.95, 95% CI 1.67–5.20, *p* < 0.001) and subretinal fluid at baseline (OR 3.17, 95% CI 1.62–6.18, *p* = 0.002). The sensitivity and specificity of the baseline OCT features were: baseline intraretinal cysts; (sensitivity: 54.2%, specificity: 65.2%) and subretinal fluid; (sensitivity: 85.5%, specificity: 28.3%).

**Table 3:** Predictors of persistent disease activity

	Univariate Odds ratio (95% C.I.)	P value	Multivariate Odds ratio (95% C.I.)	P value
Age (years)	1.01 (0.98–1.04)	*P* = 0.459	–	*P* = 0.281
**Gender (%)**	**0.62 (0.37–1.04)**	***P*** **= 0.067**	**0.54 (0.32–0.93)**	***P*** **= 0.025**
Race (%)	0.82 (0.51–1.33)	*P* = 0.427	–	*P* = 0.385
Diagnosis (%)	0.94 (0.51–1.73)	*P* = 0.838	–	*P* = 0.541
VA	1.33 (0.70–2.51)	*P* = 0.385	–	*P* = 0.932
Central retinal thickness	1.00 (1.00–1.01)	*P* = 0.279	–	*P* = 0.314
Intraretinal fluid	1.14 (0.67–1.94)	*P* = 0.626	–	*P* = 0.114
**Intraretinal cysts**	**2.22 (1.32–3.73)**	***P*** **= 0.002**	**2.95 (1.67–5.20)**	***P*** **= < 0.001**
**Subretinal fluid**	**2.32 (1.25–4.30)**	***P*** **= 0.007**	**3.17 (1.62–6.18)**	***P*** **= 0.002**
Pigment epithelial detachment	0.77 (0.15–4.06)	*P* = 0.412	–	*P* = 0.425

**Fig. 1:**
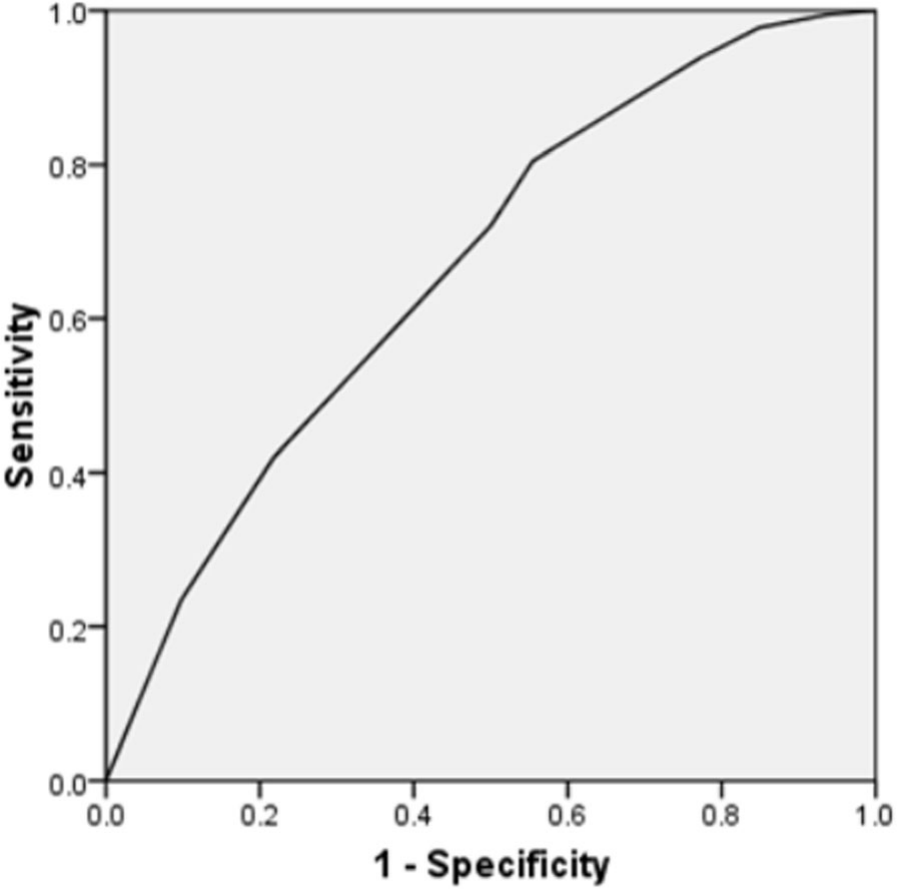
Receiver operating characteristic (ROC) curve. The ROC curve had an area under the curve of 0.666 (95% CI 0.60–0.74)

Using these factors, the receiver operating characteristic ROC curve is shown in Fig. 1, with an area under the curve of 0.666 (95% CI 0.60–0.74).

### OCT features of disease activity

At baseline, all 281 patients had at least one feature of fluid on OCT, which included subretinal fluid (229 eyes, 81.5%), intraretinal fluid (188 eyes, 66.9%) and intraretinal cysts (135 patients, 48.0%). After 3 consecutive anti-VEGF injections, 163 patients (58.0%) were found to have at least one feature of fluid on OCT. The proportion of features of disease activity on OCT at 12 weeks were subretinal fluid: 103 eyes (36.7%), intraretinal fluid: 95 eyes (33.8%), and intraretinal cysts: 74 eyes (26.3%).

The presence of intraretinal cysts and intraretinal fluid at baseline significantly affected mean final BCVA. Among those with intraretinal cysts at baseline, mean final BCVA was 0.69 compared to 0.43 for those without cysts (*p* < 0.001). Patients with intraretinal fluid at baseline had significantly worse final BCVA compared to those without intraretinal fluid (0.62 vs. 0.43, *p* < 0.001). There were no significant differences in VA at 3 months among patients with or without subretinal fluid at baseline (mean VA 0.56 vs. 0.55, *p* = 0.901).

The proportion of patients with persistent intraretinal cysts at 3 months was significantly lower among patients receiving ranibizumab or aflibercept compared to the group receiving bevacizumab (17.0% vs. 32.5%, *p* = 0.004). Similarly, the proportion of patients with persistent intraretinal fluid at 3 months was significantly lower among patients receiving ranibizumab or aflibercept compared to the group receiving bevacizumab (25.0% vs. 39.6%, *p* = 0.011). There was no difference in the proportion of patients with persistent subretinal fluid between the treatment groups.

## Discussion

In this study of real-world outcomes among a cohort of patients with nAMD and PCV, we found that 66.1% of patients still had persistent disease activity after the initial 3 loading doses of anti-VEGF agents. The significant predictive factors for persistent disease activity at 3 months were male gender, and the presence of intraretinal cysts and subretinal fluid on OCT at baseline. These factors may serve as biomarkers that predict the likelihood of persistent activity after an initial loading dose of anti-VEGF agents.

The features of disease activity detected using OCT decreased by 41.6% (from 100% to 58.0%) after treatment with 3 consecutive loading doses of anti-VEGF agents. Among OCT features, intraretinal cysts and intraretinal fluid at baseline significantly affected the final BCVA, whereas the presence of subretinal fluid did not.

The majority of large randomized clinical trials report their primary study outcomes at 6 or 12 months, with some follow-ups extending to 24 months or longer. There are, however, few studies that focus in detail on the interim outcomes and proportion of disease activity at 3 months, after the initial 3 loading dose of anti-VEGF agents. Similarly, studies reporting factors affecting clinical outcome typically examine the effects of the various risk factors at 12 months, with few studies reporting the factors affecting the initial 3-month outcomes.

It is useful to examine the factors affecting the early treatment response, because studies have demonstrated that the initial treatment response to anti-VEGF treatment in nAMD could be helpful to predict the long term visual and anatomical outcomes of treatment. Bloch et al. [4] reported that visual acuity at month 3 was the strongest predictor of final visual acuity at month 12. Predictive factors for BCVA ≤35 letters after 12 months were BCVA ≤35 letters at baseline and month 3 (*p* < 0.0001) while BCVA ≥70 letters at month 12 was associated with BCVA ≥70 letters at baseline and month 3 (*p* < 0.001) and with total lesion size < 4 disc areas (*p* = 0.0147). Similarly, Lai et al. [5] reported that persistence of intraretinal cysts at month 12 was associated with the persistence of non-resolved cysts at month 3, after the initial loading dose.

Baseline morphological features seen on OCT have been shown to predict final outcomes in some post hoc analysis of clinical trials. In a post hoc analysis of the VIEW study [19], morphologic features on OCT at baseline such as intraretinal cysts, subretinal fluid and pigment epithelial detachments significantly correlated with change in visual acuity at week 52. It was found that intraretinal cysts and pigment epithelial detachment at baseline were associated with reduced final vision gain, while subretinal fluid confers a protective effect and was associated with better vision gain.

Similarly, the current study has demonstrated that the features of disease activity on OCT decreased by over 40% following the initial 3 loading doses of anti-VEGF. Two specific baseline OCT features – the presence of intraretinal cysts and subretinal fluid – were predictive for persistent disease activity.

In this study, the presence of subretinal fluid at baseline did not significantly affect BCVA, which supports the findings in some studies which have suggested that the presence of persistent subretinal fluid following treatment in patients with nAMD may not adversely affect visual outcomes [19].

Interestingly, treatment with both ranibizumab and aflibercept resulted in lower proportion of eyes with persistent intraretinal cysts and intraretinal fluid at 3 months, compared to the group treated with bevacizumab, suggesting that these drugs may have a stronger treatment effect in the initial treatment period.

Extrapolating the results from major randomized clinical trials, the gain in BCVA after 3 loading doses ranges from 3.0 to 7.5 [20–22]. In our study, we observed a 3 letter gain in the cohort of nAMD and PCV patients. Hence, the visual gain in this study appears to be at the lower end of the reported range for several clinical trials on nAMD and PCV.

Among real-world studies, the AURA study reported a gain of 4.0 letters at week 12 among patients who received a loading dose [23]. A retrospective study of 279 patients with nAMD treated with ranibizumab reported a gain of 4.7 letters at 3 months compared to baseline [4].

Similarly, “real world” anatomical results on OCT are less ideal than that reported in clinical trials. In our cohort, CSFT decreased by a mean of 93.3 μm at week 12. In the CATT and PIER studies, CSFT was reported to decrease by approximately 70 to 175 μm at week 12 in nAMD patients [20, 21]. In the EVEREST 2 and PLANET studies, CRT decreased by approximately 75 to 130 μm in PCV patients [12, 24].

It is recognized that the clinical outcomes achieved in major clinical trials are usually better compared to real-world studies. The potential reasons include lower number of injections in real-world studies due to difficulty in scheduling injections, loss to follow up, lack of compliance and heavy patient load.

The strengths of this study include the inclusion of a large number of patients from an Asian population. This allows us to evaluate and understand the real-world outcomes of anti-VEGF outside an artificially controlled setting of a clinical trial. The risk factors identified for persistent disease activity may also be of use in the prognostication of patients. These findings can help clinicians identify potential high-risk patients for whom a more rigorous treatment regimen and follow-up can be planned. Furthermore, there is a good balance of nAMD and PCV cases in the local population that allows us to investigate the outcome of anti-VEGF monotherapy in PCV cases. Grading of images both for confirmation of diagnosis of nAMD and PCV, as well as the presence of disease activity, were performed by experienced graders from a Central Reading Center and using standardized diagnostic and grading protocols.

This study is not without limitations. The inherent limitations of a retrospective study such as selection bias due to non-randomised treatment, and potential loss to follow up.

## Conclusion

Baseline OCT features such as the presence of intraretinal cysts and subretinal fluid are predictive of persistent disease activity. This may aid clinicians in identifying patients at risk for closer monitoring and more aggressive therapy.

## Data Availability

The datasets generated and/or analysed during the current study are not publicly available due to restrictions under the Personal Data Protection Act 2012, Singapore, but are available from the corresponding author on reasonable request.

## Abbreviations

VEGF: Vascular endothelial growth factor
nAMD: Neovascular age-related macular degeneration
OCT: Optical coherence tomography
BCVA: Best-corrected visual acuity
OR: Odds ratio
CI: Confidence interval
AMD: Age-related macular degeneration
PCV: Polypoidal choroidal
FA: Fluorescein angiography
ICGA: Indocyanine green angiography
CSFT: Central subfield retinal thickness
VA: Visual acuity
ETDRS: Early Treatment Diabetic Retinopathy Study
